# Endoscopic ultrasound guided fine needle biopsy (EUS-FNB) from peritoneal lesions: a prospective cohort pilot study

**DOI:** 10.1186/s12876-021-01953-9

**Published:** 2021-10-24

**Authors:** Pradermchai Kongkam, Theerapat Orprayoon, Sirilak Yooprasert, Nakarin Sirisub, Naruemon Klaikaew, Anapat Sanpawat, Shahram Safa, Wiriyaporn Ridtitid, Pinit Kullavanijaya, Rungsun Rerknimitr

**Affiliations:** 1 Excellence Center for Gastrointestinal Endoscopy, King Chulalongkorn Memorial Hospital, Thai Red Cross Society, and Division of Gastroenterology, Department of Medicine, Faculty of Medicine, Chulalongkorn University, Bangkok, Thailand; 2 Pancreas Research Unit, Department of Medicine, Faculty of Medicine, Chulalongkorn University, Bangkok, Thailand; 3 Department of Obstetrics and Gynecology, Faculty of Medicine, Chulalongkorn University and King Chulalongkorn Memorial Hospital, Thai Red Cross Society, Bangkok, Thailand; 4 Department of Pathology, Faculty of Medicine, Chulalongkorn University and King Chulalongkorn Memorial Hospital, Thai Red Cross Society, Bangkok, Thailand

**Keywords:** Peritoneal carcinomatosis, Carcinomatosis peritonei, Omental cake, Omentum, Endoscopic ultrasound (EUS), Endoscopic ultrasound-guided fine needle aspiration (EUS-FNA), Endoscopic ultrasound-guided fine needle biopsy (EUS-FNB), Abdominal paracentesis, Ascites, Peritoneal space, Peritoneal ligament

## Abstract

**Background:**

Diagnostic laparoscopy is often a necessary, albeit invasive, procedure to help resolve undiagnosed peritoneal diseases. Previous retrospective studies reported that EUS-FNA is feasible on peritoneal and omental lesions, however, EUS-FNA provided a limited amount of tissue for immunohistochemistry stain (IHC).

**Aim:**

This pilot study aims to prospectively determine the effectiveness of EUS-FNB regarding adequacy of tissue for IHC staining, diagnostic rate and the avoidance rate of diagnostic laparoscopy or percutaneous biopsy in patients with these lesions.

**Methods:**

From March 2017 to June 2018, patients with peritoneal or omental lesions identified by CT or MRI at the King Chulalongkorn Memorial Hospital, Bangkok, Thailand were prospectively enrolled in the study. All Patients underwent EUS-FNB. For those with negative pathological results of EUS-FNB, percutaneous biopsy or diagnostic laparoscopy was planned. Analysis uses percentages only due to small sample sizes.

**Results:**

A total of 30 EUS-FNB passes were completed, with a median of 3 passes (range 2–3 passes) per case. For EUS-FNB, the sensitivity, specificity, PPV, NPV and accuracy of EUS-FNB from peritoneal lesions were 63.6%, 100%, 100%, 20% and 66.7% respectively. Adequate tissue for IHC stain was found in 25/30 passes (80%). The tissues from EUS results were found malignant in 7/12 patients (58.3%). IHC could be done in 10/12 patients (83.3%). Among the five patients with negative EUS results, two underwent either liver biopsy of mass or abdominal paracentesis, showing gallbladder cancer and adenocarcinoma. Two patients refused laparoscopy due to advanced pancreatic cancer and worsening ovarian cancer. The fifth patient had post-surgical inflammation only with spontaneous resolution. The avoidance rate of laparoscopic diagnosis was 58.3%. No major adverse event was observed.

**Conclusions:**

EUS-FNB from peritoneal lesions provided sufficient core tissue for diagnosis and IHC. Diagnostic laparoscopy can often be avoided in patients with peritoneal lesions.

**Supplementary Information:**

The online version contains supplementary material available at 10.1186/s12876-021-01953-9.

## Key summary

1. Summarise the established knowledge on this subject
  Diagnostic laparoscopy is the gold standard test for diagnosing the cause of omental cake
  EUS-FNA can provide cyto-pathological diagnosis of omental cake in the majority of patients but is an unproven technique for core biopsy with immunohistochemical staining.
2. What are the significant and/or new findings of this study?
  EUS-FNB can provide sufficient tissue for pathological diagnosis with immunohistochemical staining in the majority of patients with omental cake.

## Introduction

Peritoneal or omental lesions including peritoneal thickening, peritoneal mass or nodule, omental cake or mass can result from a variety of diseases. To unify terminologies, lesions are universally referred to as peritoneal lesions in our study. To diagnose etiologies of peritoneal lesions, several modalities including radiological findings, tissue biopsy under radiological guidance, and diagnostic laparoscopy have been used. Cross-sectional imaging findings of peritoneal lesions alone are too non-specific to provide an etiological diagnosis. Often, this method may underestimate peritoneal disease burden, since it has limitations in the detection of a small volume peritoneal implants or ascites [1]. Presumptive diagnosis of malignant peritoneal lesions in known primary cancer is not always correct as these lesions can arise from a second primary peritoneal malignancy in 10% of cases [2]. A definite diagnosis of peritoneal lesions is hence required for precise clinical management.

The limitations of current procedures underscore the need for biopsy confirmation of peritoneal lesions. The rapid advancements in cancer therapy along with greater focus on patient outcomes demand an ever-increasing need for precise staging and exact diagnosis. Immunohistochemical (IHC) staining and molecular analysis, which can be more easily examined with core tissue than cytology, has been increasingly requested by oncologists. Diagnostic laparoscopy is considered the gold standard in the diagnosis of peritoneal lesions with a sensitivity 86% and an ability to provide core tissue for IHC staining [3]. Nevertheless, it is an invasive surgical procedure. A simple and low-cost bedside procedure like abdominal paracentesis may also be helpful in the diagnosis of malignancy-related ascites. Unfortunately, the sensitivity of ascitic fluid cytology for detecting malignancy is much lower at 57–67.1% and paracentesis does not provide core tissue [4–8]. Paracentesis is also not feasible in cases without ascites, which accounts for two-thirds of patients with peritoneal carcinomatosis [9]. While CT-guided percutaneous peritoneal lesions biopsy has also been reported with a sensitivity of 89.5%, this procedure necessitates radiation exposure and is problematic for deeply located target lesions in the abdomen [10].

More recently, EUS-guided fine needle aspiration (EUS-FNA) of peritoneal lesions has been shown to be feasible, with a sensitivity between 90–100% in several case reports as well as in a large retrospective study of 98 patients by Levy et al. [11–13]. Nevertheless, it is not practical to use tissue from EUS-FNA for IHC staining due to the relatively small volume of tissue collected compared to a EUS-guided fine needle biopsy (EUS-FNB) [14]. To the best of our knowledge, no study of EUS-FNB for diagnosis of peritoneal lesions has been undertaken. This prospective study aimed to fill this research gap and evaluate the adequacy of tissue for immunohistochemical staining of EUS-FNB from peritoneal lesions and determine the diagnostic yield.

## Methods

### Patients

From March 2017 to June 2018, consecutive patients aged > 18 years with peritoneal lesions identified by CT or MRI of the abdomen at King Chulalongkorn Memorial Hospital, Bangkok, Thailand were enrolled in the study. All patients signed a consent form before undergoing the procedure. CT or MRI revealed features of peritoneal lesions including peritoneal nodules, plaques or sheets of soft tissue in the sub-hepatic area, anterior abdominal wall, paracolic gutter and cul-de-sac, stranding around and thickening of the omentum (omental cake), and thickening, stranding and distortion of the mesentery with or without presence of ascites. The exclusion criteria included uncorrectable coagulopathy, esophageal obstruction, undetectable target lesions by EUS, poor ECOG score (> 2) signifying being unfit for EUS procedure, intervening tumor in puncture site, and pregnancy.

Patient baseline characteristics including age, sex, comorbidities, previous diagnosis and treatment of malignancy, imaging features of abdomen, EUS findings of lesions, number of passes, procedural time, results of pathology and complications were recorded and analyzed.

This study used a prospective design to evaluate the adequacy of tissue for immunohistochemical staining of EUS-FNB and the diagnostic yield of EUS-FNB in patients with undiagnosed peritoneal lesions. The study was approved by the Institutional Review Board of Chulalongkorn University in Bangkok, Thailand with IRB number 422/60. The clinical trial number is TCTR 20170424001. We confirmed that all methods were carried out in accordance with relevant guidelines and regulations in the ethical approval and consent to participate subsection of declaration.

### Endoscopic techniques

Prophylactic antibiotics were administered before performing the procedure. An EUS examination of the pancreas and other intra-abdominal organs was performed using a linear echoendoscope (EG-3270UK: Pentax Corporation and Ultrasound Scanner PREIRUS: Hitachi). Once identified, EUS-FNB from the peritoneal lesions was performed with a 20-gauge needle (EchoTip ProCore 20 gage needle; Cook Medical, Limerick, Ireland). This was done in order to obtain sufficient tissue for pathology, not only for cytology. Specimens obtained from EUS-FNB were placed on a glass slide for gross visualization to evaluate adequacy of specimens as described by Iwashita et al. [15]. Adequacy was scored following a protocol published by Wang et al. [16] EUS-FNB was repeated up to four times until the specimen seemed adequate by gross visualization of white matter in the specimen as previously described. (Additional file 1: Video 1) If suspicious lymph nodes or primary lesions were identified, these lesions were also sampled after EUS-FNB of the peritoneal lesions. The results of the biopsy from these lymph nodes or lesions were used as part of the final diagnosis. To perform EUS-FNB from target peritoneal lesions, endosonographer avoided intervening tumors or other organs such as the colonic wall.

### Diagnosis

Pathology results were interpreted later at the pathological laboratory room by a gastrointestinal pathologist. Specimens were evaluated for tissue adequacy and cytopathological results. Adequacy of specimens was graded by a score previously described [16]. We used the result of the surgical or EUS-FNB pathology as the reference standard. Only a definite pathological interpretation of malignancy was considered positive, while all other suspicious or atypical types were reported as negative. If the pathology result was positive, the final diagnosis was malignant peritoneal lesions. If the result was negative or suspicious, diagnostic laparoscopy or percutaneous biopsy was planned. This approach is similar to EUS nodal staging in patients with lung cancer where patients with negative EUS-FNA from mediastinal lymph node are consequently advised to undergo mediastinoscopy [17]. In patients deemed unfit for or denied further diagnostic tests, a minimum six-month follow-up for clinical symptoms, radiology and blood tests was mandatory before making a presumptive diagnosis. Diagnostic yield of EUS-FNB and adequacy of tissue for immunohistochemical staining of EUS-FNB from peritoneal lesions were calculated as the primary endpoint.

**Fig. 1:**
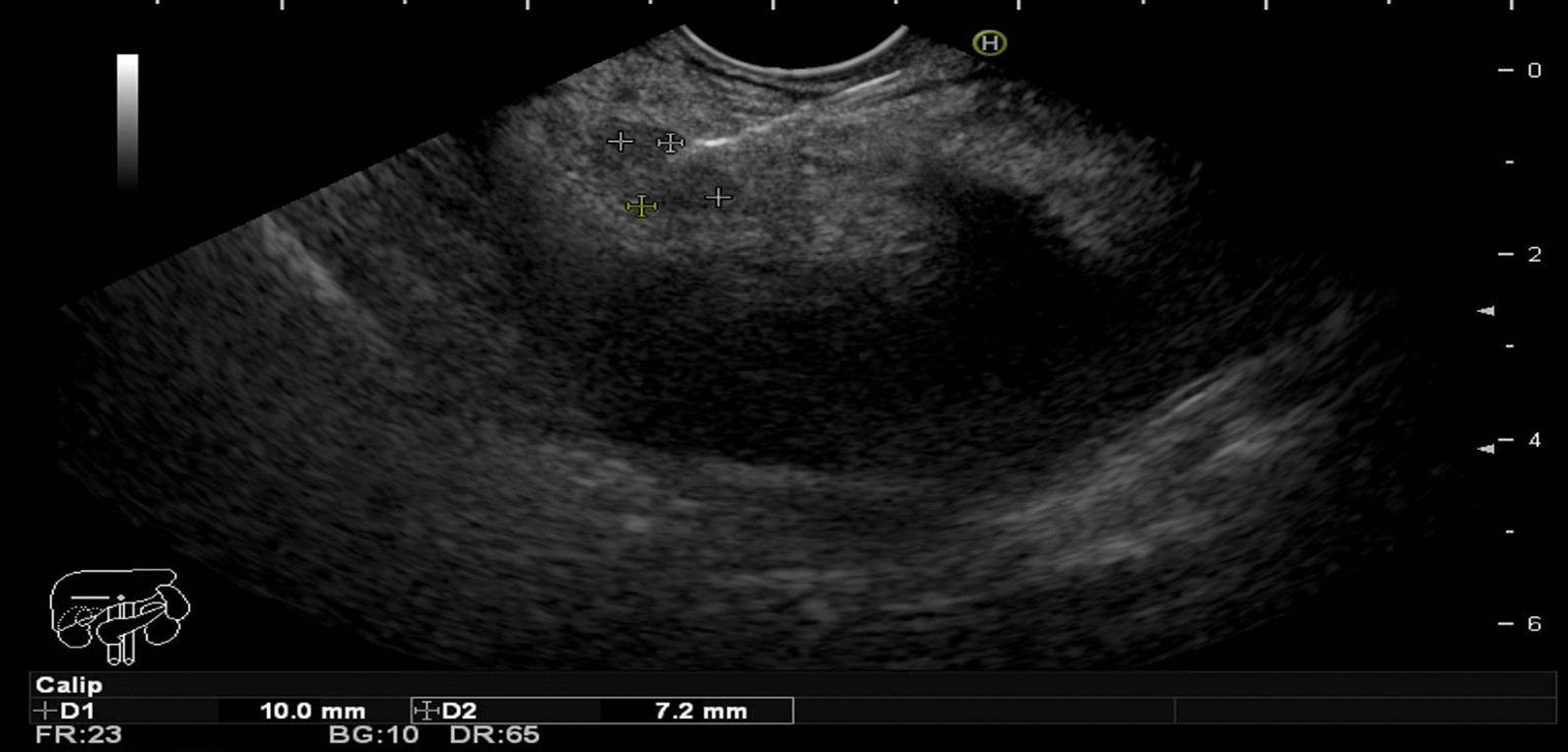
Demonstrated a hypoechoic lesion in hyperechoic thickening peritoneum. The lesion was biopsy with a 20-gauge needle (EchoTip ProCore 20 gage needle; Cook Medical, Limerick, Ireland)

**Fig. 2:**
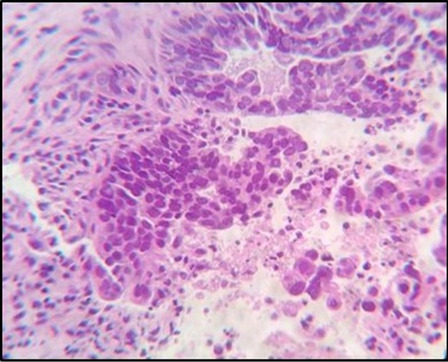
Showed peritoneal tissue from a 20G endoscopic ultrasound guided fine needle biopsy needle. Histochemical staining showed metastatic tumor sheet

**Fig. 3:**
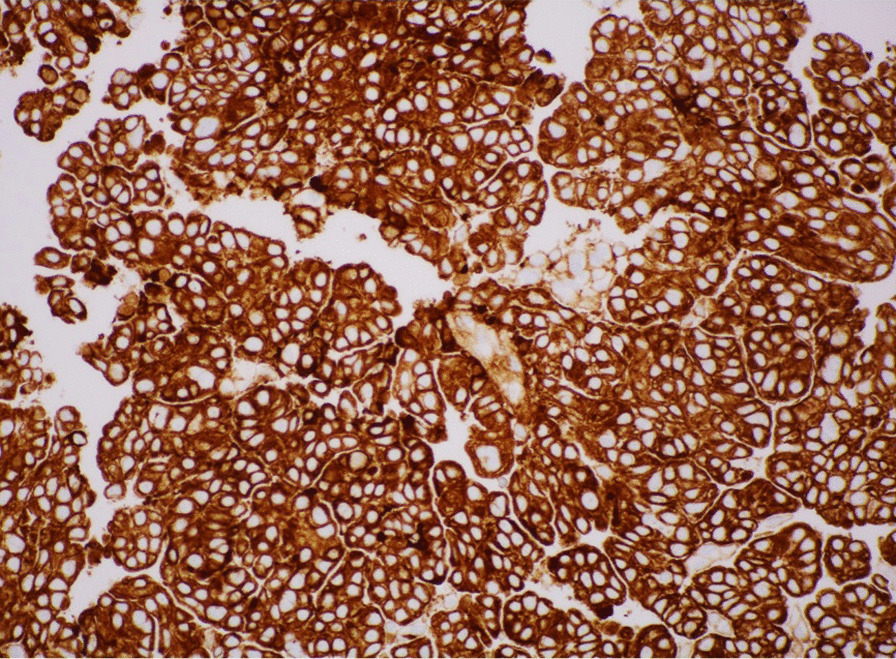
Showed positive results of immunohistochemical staining from tissue obtained from a 20G endoscopic ultrasound guided fine needle biopsy needle

A definite diagnosis on the etiology of the peritoneal lesion was made by cytological or histological interpretation of EUS-FNB or surgical specimens (Figs. 1, 2, 3). As this was a prospective study, all patients were encouraged to have a pathological diagnosis. In cases where no further intervention was done, a probable diagnosis was determined by clinical criteria and monitoring for disease progression such as increased lesion size with or without clinical deterioration, evidenced by cross-sectional imaging, or treatment response over a 6-month follow-up. This criterion of probable diagnosis is like previous EUS studies [18].

### Follow up

All patients were closely followed-up after the procedure for possible complications. Patients with positive EUS-FNB results were treated as having peritoneal malignancy. Results were sent to a multidisciplinary team. Impact on clinical management, particularly decision making for diagnostic laparoscopy from EUS-FNB results of peritoneal lesions, was recorded. Patients with negative EUS-FNB results proceeded to diagnostic laparoscopy. Those who did not consent to laparoscopy were advised to undergo percutaneous biopsy as a second option. Patients who refused both options were followed-up clinically.

### Statistical analysis

Continuous variables were expressed as mean ± SD. Categorical variables were reported as frequency (%). For the diagnostic study, sensitivity, specificity, accuracy, positive predictive value (PPV) and negative predictive value (NPV) were calculated. Chi-square and T-test were used when appropriate. SPSS version 22.0 was used for analysis.

## Results

### Patient characteristics

From March 2017 to June 2018, 23 patients met inclusion criteria. Eleven of them were excluded with reasons including poor performance status (n = 4), undetectable lesions during EUS (n = 4), intervening tumor in puncture site (n = 1), uncontrolled infection (n = 1), and esophageal obstruction (n = 1). Eventually, 12 patients with CT findings suspicious for peritoneal lesions were enrolled in the study. Patients had a mean age of 62.9 ± 9 years with an equal number of males and females. The presenting symptoms included abdominal pain (n = 6; 50%), weight loss (n = 4; 33.3%) and abdominal distension (n = 2; 16.7%). The CT findings found in the patients were soft tissue nodules/mass deposit in peritoneum (n = 11; 91.7%), ascites (n = 11; 91.7%), omental cake (n = 4; 33.3%), and mesenteric stranding (n = 2; 16.7%). Number of abnormal CT findings in 12 patients were: 1 abnormal CT finding (n = 2), 2 CT findings (n = 6); 3 CT findings (n = 2); and 4 CT findings (n = 2). Patient’s demographic data and clinical presentation are shown in Table 1. Ascites was demonstrated by CT in 11 from 12 patients (91.7%). Abdominal paracentesis was feasible in 9 patients (75%). Paracentesis was not performed in 3 patients because of insufficient amount of ascites (Table 2).

**Table 1:** Demographic data and clinical presentation of study patients

		EUS-FNB N = 12 (%)*
Gender	Male	6 (50)
	Female	6 (50)
Age, years (mean + SD)		62.9 ± 9
Presenting symptoms	Weight loss	4 (33.3)
	Abdominal Pain	6 (50)
	Abdominal Distention	2 (16.7)
	Jaundice	0 (0)
CT findings^#^	Soft tissue nodules/mass deposit in peritoneum	11 (91.7)
	Ascites	11 (91.7)
	Omental cake	4 (33.3)
	Mesenteric stranding	2 (16.7)
EUS findings	Thickened hyperechoic omental cake	1 (8.3)
	Hypoechoic nodules/deposit in peritoneum or omentum	11 (91.7)

*All values reported as n (%), except for age

^#^Small ascites defined by shortest thickness of ascites from abdominal wall < 3 mm in CT or MRI

**Table 2:** Detection of ascites and performance of abdominal paracentesis

		EUS-FNB (N = 12 (%))
Ascites	Detected on CT	11 (91.7)
	Detected on EUS	12 (100.0)
Abdominal Paracentesis	Feasible	9 (75.0)
	Not feasible	3 (25.0)

One from 9 patients who underwent abdominal paracentesis had a positive malignant cell from ascitic fluid

### Diagnosis

**Fig. 4:**
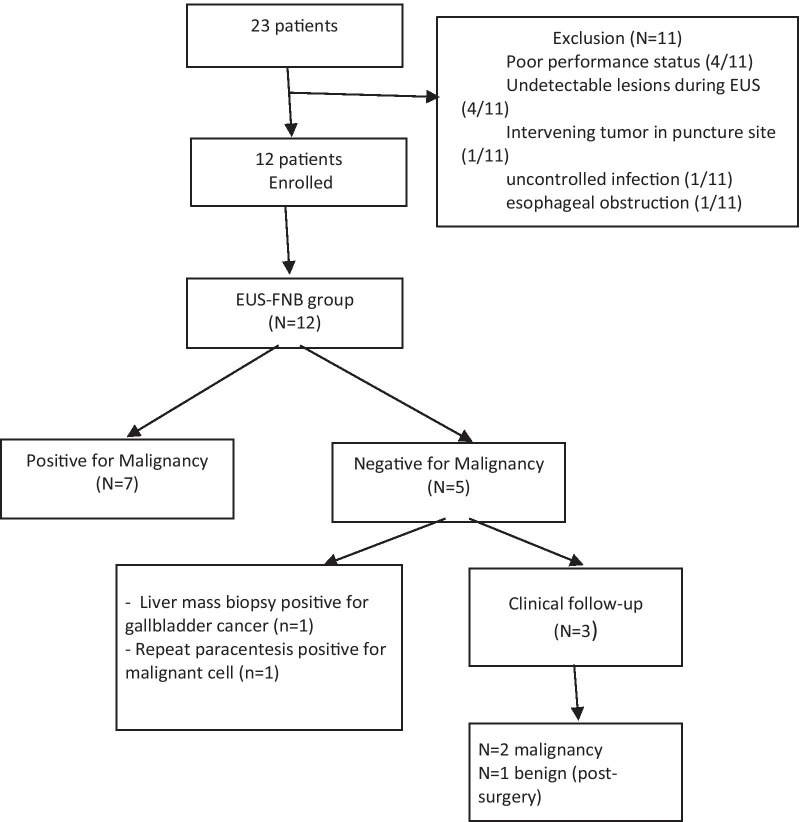
This flow chart summarized overall results of all enrolled patients

Malignant peritoneal lesions were found in 11 patients with one patient having benign lesions. Procedures used in malignancy diagnoses included cytology from pathology by EUS-FNB (n = 7), repeat abdominal paracentesis (n = 1), percutaneous liver biopsy (n = 1), and adequate follow up with evidence of disease progression over a 6-month follow-up (n = 2). In a benign peritoneal lesion, etiology was identified post-surgery (n = 1). In the benign peritoneal lesion, the patient received follow up for more than 1 year (Fig. 4).

### EUS-FNB

All FNB needle punctures were inserted through the stomach. There were a total of 30 passages with each patient receiving two or three passes. The tissues from EUS results found malignancy in 7 patients. The laparoscopic avoidance rate is 7/12 (58.3%). IHC stain was achieved in 10/12 patients (83.3%). The sensitivity, specificity, PPV, NPV and accuracy of EUS-FNB from peritoneal lesions were 63.6%, 100%, 100%, 20% and 66.7%, respectively (Fig. 1). The positive EUS-FNB finding for malignant lesions occurred in 17 passages with nearly all from hypoechoic lesions in 16/17 lesions (94.1%) and one from hyperechoic lesions.

Definite pathological diagnosis of malignant peritoneal lesion from the EUS-FNB group was made in 9/12 patients. Seven patients were diagnosed from EUS-FNB specimens. One patient was diagnosed with adenocarcinoma with repeated abdominal paracentesis. Another patient was sent for liver biopsy and diagnosed with gallbladder carcinoma. The other three patients were provided clinical follow-up. One patient died after 1 month of follow-up. One patient experienced clinical worsening with an increase of ascites. Another patient clinically resolved and was diagnosed with post-operative inflammation (Table 3).

**Table 3:** Final diagnosis in all patients

	EUS-FNB N = 12 (%)*
Pancreatic cancer	4 (33.3)
Gallbladder cancer	1 (8.3)
Gynecologic malignancy	3 (25)
Cholangiocarcinoma	1 (8.3)
Hepatocellular carcinoma	1 (8.3)
Carcinoma unknown primary	1 (8.3)
Benign disease	1 (8.3)

*All values reported as n (%)

One EUS-FNB patient had an incident of abdominal pain which responded to analgesic drug regimen. Another patient experienced obscure anemia needing two units of red cell transfusion which was classified as moderate severity according to a lexicon for endoscopic adverse events [19].

## Discussion

EUS-FNA and EUS-FNB have been world widely used for tissue diagnosis of solid pancreatic lesions over the last 2 decades. Varying designs and diameters of EUS-FNA and EUS-FNB needles have been developed and tested to be used properly in different kinds of lesions and circumstances. For example, a meta-analysis of EUS-FNA from solid pancreatic lesions showed no superiority of 25- over 22-gauge needles [20]. In addition to solid pancreatic lesions, several studies have been done and demonstrated that EUS-FNB can provide high diagnostic yield in various targets e.g., subepithelial lesions, liver parenchyma, etc. [21, 22]. Nowadays, EUS technique has been expanded to more therapeutic options [23].

The design of this current study is to evaluate the clinical use of EUS-FNB for peritoneal lesions in similar fashion to the use of EUS for mediastinal staging of lung cancer where diagnostic mediastinoscopy is performed only in cases of negative EUS-FNB. Results from this present study show an impressive avoidance rate of diagnostic laparoscopy or percutaneous biopsy of 58.3%. Clinicians who are treating patients with peritoneal lesions should consider EUS-FNB as a first-line test before diagnostic laparoscopy. This guidance is supported by results of previous studies that used EUS-FNA for diagnosing etiologies of peritoneal lesions including Levy et al., which demonstrated that EUS-FNA can detect peritoneal carcinomatosis better than CT/MRI with high sensitivity, specificity, and accuracy rate at 91%, 100%, and 94%, respectively, as compared to 28%, 85%, and 47%, respectively, for CT/MRI. In a retrospective study of 12 patients, Rana et al. found that EUS-FNA from peritoneal lesions could be performed safely and was able to diagnose malignant and benign peritoneal nodules in 83% (10/12) of patients [24].

Most patients with peritoneal lesions either due to benign or malignant causes produce ascites detectable by CT, which leads to abdominal paracentesis as a conventional diagnostic test. However, in two large retrospective studies, smaller amounts of ascites were undetected by CT including 6.5% (52/798) and 21.8% (12/55) [6, 11]. This current study confirmed this finding. This suggests that for patients with small amounts of ascites, even with a negative CT, EUS should be performed to confirm absence or presence of ascites because ascites can be an important diagnostic clue, and in malignant conditions can upstage the disease. Moreover, diagnosis of radiographic occult ascites in patients suspected for peritoneal carcinomatosis resulted in more accurate cancer staging and determination of resectability status [25]. Malignancy-related ascites account for about 7% of cases of ascites, with two-thirds of these patients having peritoneal carcinomatosis, which underscores the need for an accurate diagnosis to guide clinical management [26, 27].

In patients with peritoneal lesions and ascites with a negative result of abdominal paracentesis, diagnostic laparoscopy is often the next step for making a diagnosis of etiology. Unfortunately, diagnostic laparoscopy is typically an inpatient procedure requiring admission with a complication rate of 2–3% [28]. Results from this current study showed that EUS-FNB from peritoneal lesions is feasible and effective for diagnostic purposes without any serious complication. In addition, it is an outpatient procedure requiring no intensive post-procedural care and general anesthesia. Clinical application from results of this study suggests that EUS-FNB for peritoneal lesions might be considered as an initial diagnostic test before laparoscopy.

Regarding the endoscopic ultrasound findings of peritoneal lesions, Levy et al. stated that “most malignant-appearing peritoneal anomalies were solid, hypoechoic masses, thickening or nodularity in the peritoneum and/or omentum.” This observation was also noted in our study where most cases with positive results were taken from hypoechoic peritoneal lesions. Based on the findings from these two studies, we suggest endosonographers performing the EUS-FNB of peritoneal lesions to select hypoechoic lesions as the target. However, with a limitation of reviewing all EUS imaging as a retrospective review, we did not have enough information to compare the sensitivity of EUS-FNB for hypoechoic versus hyperechoic lesions. Future systematic study is needed to clarify this point.

Although EUS-FNB has demonstrated a high diagnostic accuracy rate even without rapid on-site evaluation from solid pancreatic lesions. In this current study, the sensitivity of EUS-FNB for peritoneal lesions was only 63.6% which was relatively low. This might be explained by the different design and diameter of EUS-FNB needles. Some previous studies showed a higher tissue acquisition rate of EUS-FNB from solid pancreatic lesions in new generation end -cutting needles (e.g., Fork-tip or Franseen needle) than side-fenestrated ones [29, 30]. Another new technique of tissue sampling has been introduced as through-the-needle microforceps biopsy (TTNB). A recent meta-analysis showed the sample adequacy of TTNB in pancreatic cyst was 85.3%. The pooled diagnostic accuracy rate, sensitivity, and specificity of TTNB were 78.8%, 82.2%, and 96.8% respectively [31]. Moreover, a study of EUS-TTNB revealed high interobserver agreement among pathologists [32]. Another recent case series demonstrated an impressive diagnostic performance for carcinomatosis peritonei [33]. In the future, it is interesting to compare diagnostic rate among different types of EUS-FNB needles with a fashion of head-to-head comparison study.

This study shows only one adverse event occurred with one patient experiencing obscure anemia needing 2 units of red cell transfusion; which was classified as moderate severity [19]. In comparison with the diagnostic laparoscopy, EUS-FNB is less invasive and an out-patient procedure but it is still too early to compare adverse event rate because the number of cases in this current study is too small. Nevertheless, EUS-FNB usage for peritoneal lesions has become an interesting alternative diagnostic tool compared with diagnostic laparoscopy.

The limitations of this present study include a small number of patients, very few benign cases, and no control group. These limitations can only be overcome with a larger number of patients with higher number of benign cases as a control group.

## Conclusion

This current prospective study confirmed that EUS-FNB of peritoneal lesions appears to be a technically feasible, safe, minimally invasive alternative for tissue diagnosis. The best type of lesion to produce a high yield is a hypoechoic lesion. Moreover, EUS-FNB was found to have a high rate of tissue acquisition adequacy for immunohistochemistry. The technique can avoid more invasive diagnostic laparoscopy in most patients.

## Supplementary Information

**Additional file 1**. Video 1: This Video demonstrated a peritoneal lesion being sampled with a 20-gauge needle (EchoTip ProCore 20 gage needle; Cook Medical, Limerick, Ireland).

## Data Availability

The datasets used and/or analyzed during the current study are available from the corresponding author on reasonable request.

## Abbreviations

EUS: Endoscopic ultrasound
EUS-FNB: Endoscopic ultrasound-guided fine needle biopsy
EUS-FNA: Endoscopic ultrasound-guided fine needle aspiration
IHC: Immunohistochemistry stain
CT: Computed tomography
MRI: Magnetic resonance imaging
SD: Standard deviation
PC: Peritoneal carcinomatosis
PPV: Positive predictive value
NPV: Negative predictive value
